# Transcriptomic Dynamics of Rice Varieties with Differential Cold Tolerance Under Low-Temperature Stress During Grain-Filling Stage

**DOI:** 10.3390/genes16080950

**Published:** 2025-08-11

**Authors:** Liangzi Cao, Xueyang Wang, Yingying Liu, Guohua Ding, Jinsong Zhou, Lei Lei, Liangming Bai, Yu Luo, Shichen Sun

**Affiliations:** 1 Institute of Tillage and Cultivation, Heilongjiang Academy of Agricultural Sciences, Harbin 150088, Chinahucheng229@163.com (G.D.); zjszhuo@163.com (J.Z.);; 2 Heilongjiang Rice Quality Improvement and Genetic Breeding Engineering Research Center, Harbin 150086, China; 3 Soybean Research Institute, Heilongjiang Academy of Agricultural Sciences, Harbin 150086, China; 4 Agricultural College, Northeast Agricultural University, Harbin 150000, China

**Keywords:** rice, cold stress, grain-filling stage, taste quality, starch and sucrose metabolism, SUS

## Abstract

**Background/Objectives:** Low-temperature stress during the grain-filling stage negatively affects rice grain quality and yield. Understanding the physiological and molecular mechanisms underlying cold tolerance is critical for breeding rice varieties with improved resilience. **Methods:** In this study, eight rice varieties with differential cold tolerance—LD1603, 13108, LD18, and 4-1021 (cold-tolerant) and LD3, LD4, LD121, and LD1604 (cold-sensitive)—were subjected to 17.5 °C low-temperature stress during grain filling in a naturally illuminated phytotron. Amylose and protein content, as well as taste quality, were analyzed. RNA sequencing was performed to identify differentially expressed genes and transcription factors associated with cold response. **Results:** Under low-temperature stress, amylose and protein content significantly increased in all eight varieties. The taste quality of cold-sensitive varieties declined markedly, whereas cold-tolerant varieties maintained higher and more stable taste quality values. Transcriptomic analysis revealed that key enzyme genes (*INV*, *SUS*, *HXK*, *FRK*, *amyA*, and *TPP*) in the starch and sucrose metabolism pathway were significantly upregulated in cold-tolerant varieties (LD18 and 4-1021), but suppressed in cold-sensitive varieties. Several cold-responsive transcription factors from the NAC, WRKY, AP2/ERF, MYB, and bZIP families were also identified. Weighted gene co-expression network analysis (WGCNA) further revealed hub TFs (OsWRKY1, OsWRKY24, OsWRKY53, and OsMYB4) and structural genes (*OsPAL04* and *OsCDPK7*) potentially involved in cold tolerance during grain filling. **Conclusions:** This study enhanced our understanding of the molecular response to low temperature during rice grain filling and provided candidate genes for developing cold-tolerant rice varieties through molecular breeding.

## 1. Introduction

Rice (*Oryza sativa* L.), one of the most important cereal crops globally, serves as the primary food source for nearly half of the world’s population. However, as a thermophilic and hygrophilous plant species, rice exhibits high sensitivity to low-temperature stress. With the increasing frequency of extreme weather events under global climate change, chilling injury has emerged as a major constraint for rice production worldwide. This challenge becomes particularly pronounced during the grain-filling stage, a critical developmental phase that determines grain quality formation. Exposure to low-temperature stress during this stage often prevents rice plants from achieving normal maturation or full grain development, resulting in a significantly increased proportion of immature grain and elevated amylose content, both of which severely compromise grain quality and yield potential [1,2,3]. In response to low-temperature stress, rice undergoes a series of physiological adjustments through the modulation of growth-regulating substances to maintain essential metabolic functions [4]. These adaptive responses involve multiple biochemical components, including phytohormones [5,6,7,8], soluble sugars [9,10], amino acids [11], and reactive oxygen species (ROS) and their scavenging systems [12,13]. These physiological markers have been demonstrated to correlate significantly with cold stress responses and can serve as reliable indicators for evaluating cold tolerance in rice cultivars [14]. Compared with changes at the physiological and biochemical levels, transcriptional regulation offers stronger specificity and enables more precise investigation. In recent years, RNA sequencing (RNA-seq) has been extensively applied in rice research to investigate cold-stress-responsive genes involved in transcription regulation, signal transduction, secondary metabolism, and other biological processes [15,16,17,18].

Cultivated rice comprises two major subspecies: *indica* and *japonica*. *Indica* rice is predominantly cultivated in tropical and subtropical regions characterized by higher mean annual temperatures, making it generally more sensitive to cold stress. In contrast, *japonica* rice is primarily distributed in temperate zones and high-altitude areas with lower average temperatures [19,20]. Through long-term domestication and artificial selection, *japonica* rice has developed enhanced adaptability to low-temperature growth conditions [21]. Heilongjiang Province, the leading production region for *japonica* rice in China, frequently experiences prolonged or extreme cold spells accompanied by insufficient sunlight during the grain-filling stage, significantly reducing yield and grain quality. At present, cold tolerance during the grain-filling stage has become a mandatory prerequisite for rice variety certification in Heilongjiang Province. Therefore, identifying cold-tolerance-related genes from resistant germplasm, breeding cold-tolerant varieties, and promoting genetic improvement for enhanced cold resistance are of critical importance in mitigating chilling injury during the grain-filling period.

In our preliminary screening of 51 rice accessions, four temperature-tolerant varieties (LD1603, 13108, LD18, and 4-1021) and four temperature-sensitive varieties (LD3, LD4, LD121, and LD1604) were selected based on the stability of their taste quality conducted under natural temperature fluctuations from 2018 to 2021. Using these contrasting genotypes, this study employed RNA sequencing (RNA-seq) to systematically compare transcriptomic responses to low-temperature stress during the grain-filling stage between cold-tolerant and cold-sensitive rice varieties. We aimed to identify expression pattern variations in cold-responsive genes, discover novel genes associated with cold tolerance, and unravel the molecular mechanisms underlying cold adaptation during reproductive development. Our findings enhance the understanding of cold tolerance in rice and provide a basis for the breeding of cold-resistant cultivars adapted to cooler climate regions.

## 2. Materials and Methods

### 2.1. Plant Material

In the preliminary phase of this study, we screened 51 rice accessions under natural temperature fluctuations from 2018 to 2021. Four rice varieties (LD1603, 13108, LD18, and 4-1021) that maintained relatively stable taste quality across different years were identified as cold-tolerant. In contrast, four varieties (LD3, LD4, LD121, and LD1604) that exhibited greater variation in taste quality under fluctuating temperatures were classified as cold-sensitive. These eight representative varieties were selected for further analysis (Figure S1, Table S1). Seeds of the 51 accessions were provided by the germplasm resource center of Heilongjiang Academy of Agricultural Sciences.

### 2.2. Temperature Treatments and Sampling

The experiment was conducted at the paddy field base of the Minzhu Campus, Heilongjiang Academy of Agricultural Sciences (N 45.84°, E 126.82°). All plants were initially grown in pots filled with paddy soil identical to the field soil and cultivated in the open field under standard agronomic practices for paddy rice. Transplanting was carried out on 20 May 2023, and plants were maintained under field conditions until the grain-filling stage. Eight rice varieties previously screened for their temperature response were selected for this study.

At seven days after full heading, two temperature treatments were applied (which occurred in early August, with slight variation among the different varieties): a low-temperature treatment (LT) and a control temperature treatment (CK). For both treatments, 24 plants per variety were transferred in a naturally illuminated phytotron (Koyitou, Japan). The LT group was exposed to a constant temperature of 17.5 °C, while the CK group was maintained at 25 °C for 14 days to simulate contrasting thermal conditions during the grain-filling stage [22]. After the 14-day treatment period, the pots were returned to the field and grown under natural conditions until maturity.

At maturity in early October, though the timing varied slightly among different varieties, panicles were harvested, naturally air-dried, and threshed. The grains were stored for subsequent analysis of grain quality traits and RNA sequencing. Each treatment included three biological replicates.

### 2.3. Determination of Amylose Content

Amylose content was measured using the iodine blue colorimetric method as described by Man et al. [23] with slight modifications. Briefly, 0.01 g of rice powder (passed through a 100-mesh sieve) was transferred into a 10 mL test tube. Then, 100 μL of 95% ethanol was added to aid dispersion, followed by 900 μL of 1 mol·L^−1^ NaOH. The tube was then incubated in a boiling water bath for 10 min to gelatinize the starch, cooled to room temperature, and diluted to a final volume of 10 mL with distilled water. Next, 9.2 mL of distilled water was added to a separate large test tube, followed by 500 μL of the prepared sample solution, 100 μL of 1 mol·L^−1^ acetic acid, and 200 μL of 0.2% iodine solution. The mixture was shaken thoroughly and left to stand for 10 min at room temperature. A blank control was prepared using 0.09 mol·L^−1^ NaOH in place of the sample solution. The absorbance was measured at 620 nm using a microplate reader. Amylose content was determined by referencing the standard curve.

### 2.4. Determination of Protein Content

Protein content was determined according to the semi-micro Kjeldahl method as described in the Chinese National Standard GB/T 17891-2017 (High-Quality Paddy) [24]. Briefly, the total nitrogen content of 1 g of rice powder was measured using a fully automated Foss Tecator Kjeldahl analyzer (FOSS Analytical A/S, Hillerød, Denmark). The protein content was calculated by multiplying the total nitrogen content by a conversion factor of 5.95.

### 2.5. Taste Quality Evaluation Using Descriptive Analysis (DA)

The preparation of rice samples and cooked rice was performed following the method described in the Rice Breeding Manual by Yamamoto et al. [25]. After milling, the moisture content of each rice sample was adjusted to 13.5%. A 400 g portion of the polished rice was sealed and stored at 4 °C for subsequent use. Prior to tasting, the rice was washed 4–5 times, and cooked in an electric rice cooker (MB-RE476S, Midea, Foshan, China) at a rice-to-water ratio of 1:1.3 (*w*/*w*). The rice was soaked for 30 min before cooking, and tasting evaluation was conducted 20 min after the automatic shut-off. Panelists were selected and trained following the guidelines in Yamamoto et al. [25]. The evaluation process began with a visual assessment of rice color, followed by the sensory analysis of texture attributes such as softness, stickiness, and elasticity through chewing, as described in the Chinese National Standard GB/T 5492-2008 [26] (Inspection of grain and oils: Identification of color, odour and taste of grain and oilseeds). Finally, panelists provided an overall score based on color, aroma, taste, and mouthfeel.

### 2.6. RNA-Seq and Data Analysis

Total RNA was extracted from the grains using the TRIzol reagent kit (vitrogen, Carlsbad, CA, USA) [27] according to the manufacturer’s protocol. RNA quality was evaluated using an Agilent 2100 Bioanalyzer (Agilent Technologies, Santa Clara, CA, USA) and verified by electrophoresis on an RNase-free agarose gel. Briefly, RNA-seq libraries were prepared from poly(A)-enriched mRNA using the NEBNext Ultra II RNA Library Prep Kit (New England Biolabs, Ipswich, MA, USA) following the manufacturer’s protocol. Fragmented mRNA was reverse-transcribed, adaptor-ligated, size-selected, and PCR-amplified. Qualified libraries were pooled and sequenced on an Illumina HiSeq2500 platform.

Raw sequencing data were quality-controlled using fastp (v0.23.1) [28] and aligned to the *O. sativa Japonica* Group reference genome (IRGSP-1.0) (https://www.ncbi.nlm.nih.gov/datasets/genome/GCF_001433935.1/; accessed on 20 December 2024) using HISAT2 (v2.2.1) [29]. Transcript assembly and quantification were performed using StringTie (v2.1.4) [30] and RSEM (v1.3.3) [31]. Differential expression analysis was conducted using DESeq2 (v1.34.0) [32] based on raw read counts. Genes were considered differentially expressed with the thresholds of FDR < 0.05 and |log_2_FC| > 1. Functional annotation of these genes was achieved through BLAST (2.12.0) searches against multiple databases, including NCBI non-redundant protein sequences (NR), Swiss-Prot, Gene Ontology (GO), and KEGG pathway databases. Throughout the analysis, strict quality control metrics were applied, with an average alignment rate exceeding 85% and a minimum sequencing depth of 20 million reads per sample. Three biological replicates were included for each treatment.

### 2.7. Correlation Analysis

Pearson correlation coefficients were calculated using the psych package in R (v4.4.1). Gene pairs with a Pearson correlation coefficient > 0.85 and *p* < 0.05 were selected and imported into Cytoscape software (v3.10.2) [33] for co-expression network visualization.

### 2.8. Gene Co-Expression Network Analysis

The varFilter function from the genefilter package in R was used to eliminate genes with low or non-variable expression in all samples. A gene expression matrix was constructed using weighted gene co-expression network analysis (WGCNA). The optimal soft-thresholding power was determined using the pickSoftThreshold function in the WGCNA package, with the RsquaredCut parameter set to 0.85. Module eigengenes were subsequently calculated to represent the principal component of each module, and their associations with sample traits were assessed. Finally, the gene co-expression networks were visualized using Cytoscape.

## 3. Results

### 3.1. Low-Temperature Treatment Affected the Taste Quality of Rice in Both Cold-Tolerant and Cold-Sensitive Varieties

**Figure 1:**
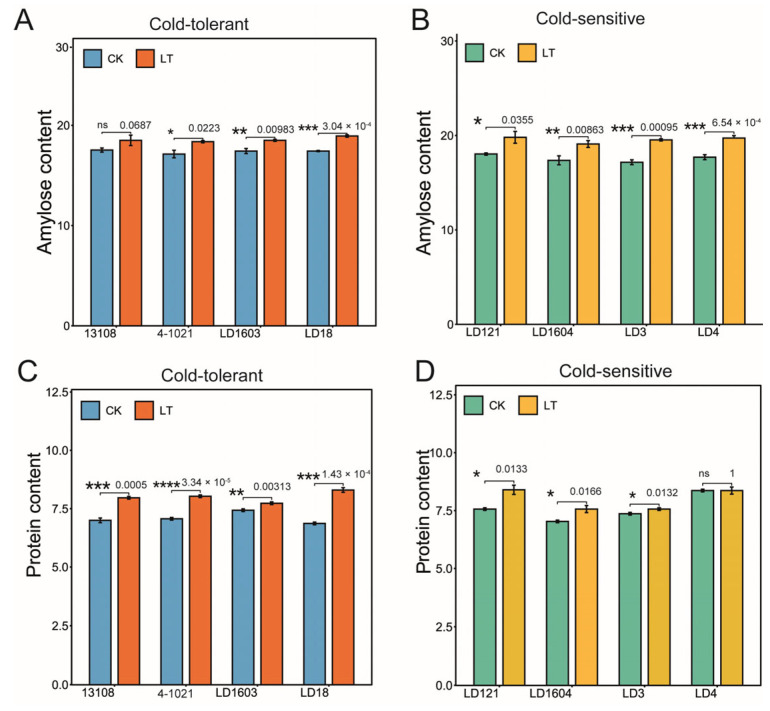

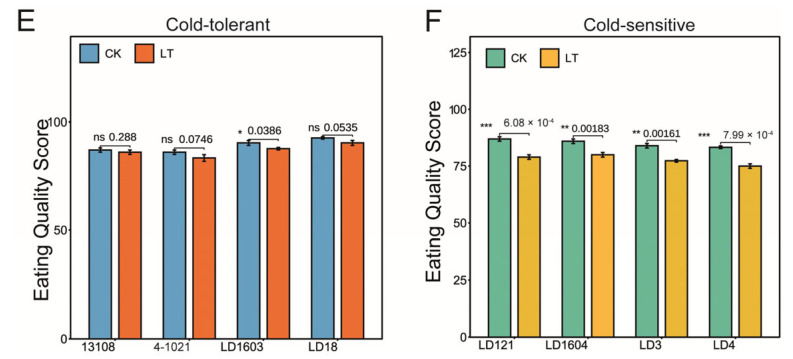
Effects of low temperature on amylose content (**A**,**B**), protein content (**C**,**D**), and taste value (**E**,**F**). The reported value is the mean ± three repeated standard errors. Asterisks indicate statistically significant differences between control (CK) and low-temperature (LT) treatments within each variety (* *p* < 0.05, ** *p* < 0.01, *** *p* < 0.001, **** *p* < 0.0001, ns = not significant). CK: control (25 °C); LT: low temperature (17.5 °C).

To investigate the effects of low-temperature stress on rice grain quality, we analyzed the changes in amylose content, protein content, and taste quality across cold-tolerant and cold-sensitive varieties. Under low-temperature conditions, compared with the control treatment, the amylose content increased significantly in all rice varieties except for the cold-tolerant variety 13108 (Figure 1A,B), indicating that the accumulation of amylose in rice was affected by low-temperature stress. Similarly, the protein content increased significantly in all varieties following low-temperature treatment, except for cold-sensitive LD4 (Figure 1C,D). Among cold-tolerant varieties, the taste quality of LD1603 decreased significantly under low-temperature treatment, while no significant difference was observed between the CK and LT groups in the other three tolerant varieties (Figure 1E). In contrast, all cold-sensitive varieties exhibited a significant reduction in taste quality following low-temperature stress (Figure 1F). These results suggest that low-temperature treatment markedly affected rice taste quality by regulating the synthesis and accumulation of amylose and protein.

### 3.2. Quality Assessment and Summary Statistics of Transcriptome Data

To investigate the molecular regulatory networks underlying low-temperature adaptation in rice, we performed RNA-seq on grains from both CK and LT samples. The experimental design comprised eight rice varieties, with three biological replicates per condition, yielding a total of 48 transcriptome libraries. After removing low-quality data, 1,948,411,032 clean reads were obtained from the 48 samples. The proportion of bases with a base quality value greater than Q20 in the clean data varied from 97.85% to 98.28%, while the proportion exceeding Q30 was between 93.68% and 94.72%. The number of reads mapped to the reference genome ranged from 29,491,623 to 44,306,105, accounting for 80.93–91.51% of the effective reads (Table S2). These results confirm the high reliability of our transcriptome dataset, ensuring robust downstream bioinformatics analyses, including differential gene expression and co-expression network construction.

### 3.3. Comparative Transcriptomic Analysis of Cold-Tolerant Rice Varieties Between CK and LT Treatments

**Figure 2:**
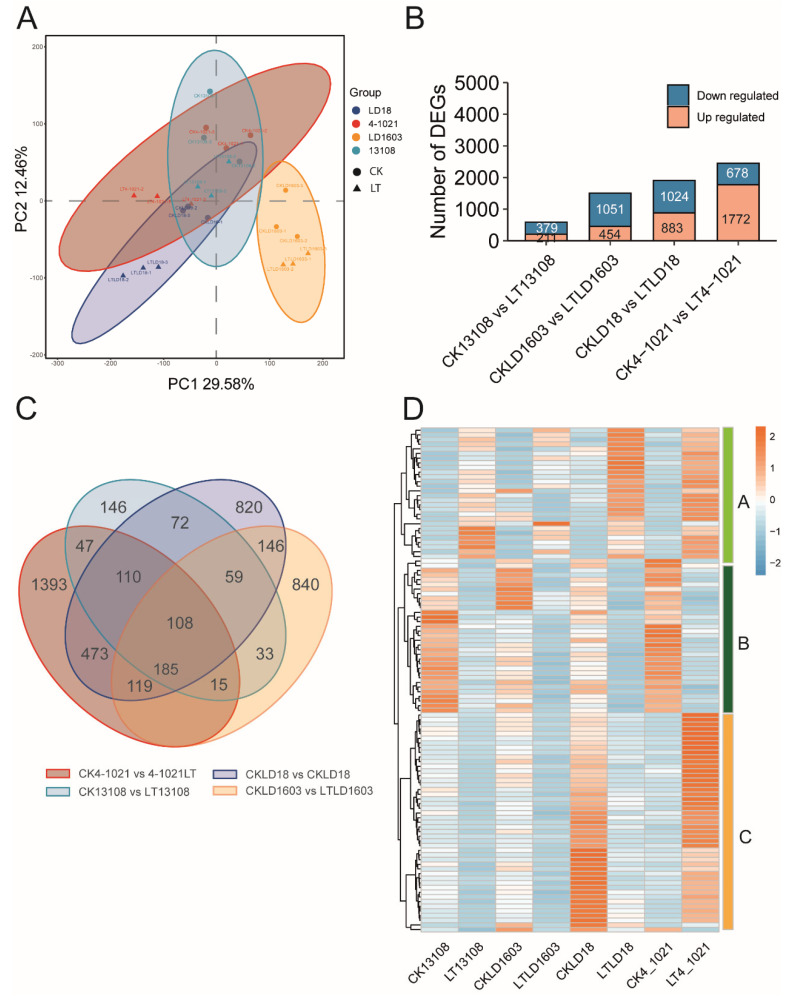
Global transcriptomic profiling of cold-tolerant rice varieties in response to low-temperature treatment. (**A**) Principal component analysis (PCA) of transcriptome data for CK and LT treatments. (**B**) Bar plot showing the number of differentially expressed genes (DEGs) identified in pairwise comparison between CK and LT treatments of the four cold-tolerant varieties. (**C**) Venn diagram illustrating the overlap of DEGs among the four comparison groups. (**D**) Heatmap showing the expression patterns of the 108 DEGs co-expressed across all four cold-tolerant varieties.

Principal component analysis (PCA) was used to analyze the transcriptomic variation among cold-tolerant samples (LD18, 4-1021, LD1603, and 13108) subjected to different treatments (Figure 2A). In the PCA plot, the samples clustered tightly within each group, while clear separation was observed between the CK and LT treatments, reflecting strong reproducibility and distinct transcriptional responses to low-temperature stress. Notably, LD1603 exhibited a distinct separation from the other three varieties. With FDR < 0.05 and |log_2_FC| > 1, a total of 4566 differentially expressed genes (DEGs) were identified in the paired comparisons between the CK and LT treatments across the four rice varieties. Specifically, 509 DEGs were identified between CK13108 and LT13108 (211 upregulated and 379 downregulated), 1505 DEGs between CKLD1603 and LT LD1603 (454 upregulated and 1051 downregulated), 1907 DEGs between CKLD18 and LTLD18 (883 upregulated and 1024 downregulated), and 2450 DEGs between CK4-1021 and LT4-1021 (1772 upregulated and 678 downregulated) (Figure 2B). In addition, Venn diagram analysis of DEGs from the four comparison groups revealed that 1393, 840, and 820 DEGs were uniquely expressed in 4-1021, LD1603, and LD16, respectively. In contrast, only 146 DEGs were specifically expressed in variety 1,3108. Moreover, 108 DEGs were commonly expressed across all four cold-tolerant varieties. These shared DEGs were classified into three main expression modules. Genes in Module A were primarily upregulated after LT treatment, especially in LD18 and 4-1021. Module B genes showed high expression in CK but were downregulated under LT treatment. Module C genes were significantly upregulated in CKLD18 and LT4-1021 (Figure 2D). These findings suggest that 4-1021 exhibited the most pronounced transcriptomic response to low-temperature treatment.

**Figure 3:**
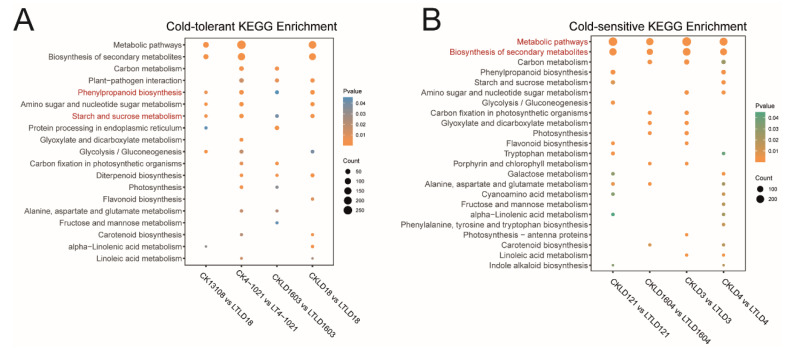
KEGG enrichment analysis of DEGs between CK and LT treatments in cold-tolerant varieties (**A**) and cold-sensitive varieties (**B**).

We performed KEGG enrichment analysis on all of the 4566 identified DEGs. The results revealed significant enrichment in phenylpropanoid biosynthesis as well as starch and sucrose metabolism (*p* < 0.05). Other pathways, such as metabolic pathways, biosynthesis of secondary metabolites, plant–pathogen interaction, and glycolysis/gluconeogenesis, were also significantly enriched (Figure 3A).

### 3.4. Comparative Transcriptomic Analysis of Cold-Sensitive Rice Varieties Between CK and LT Treatments

**Figure 4:**
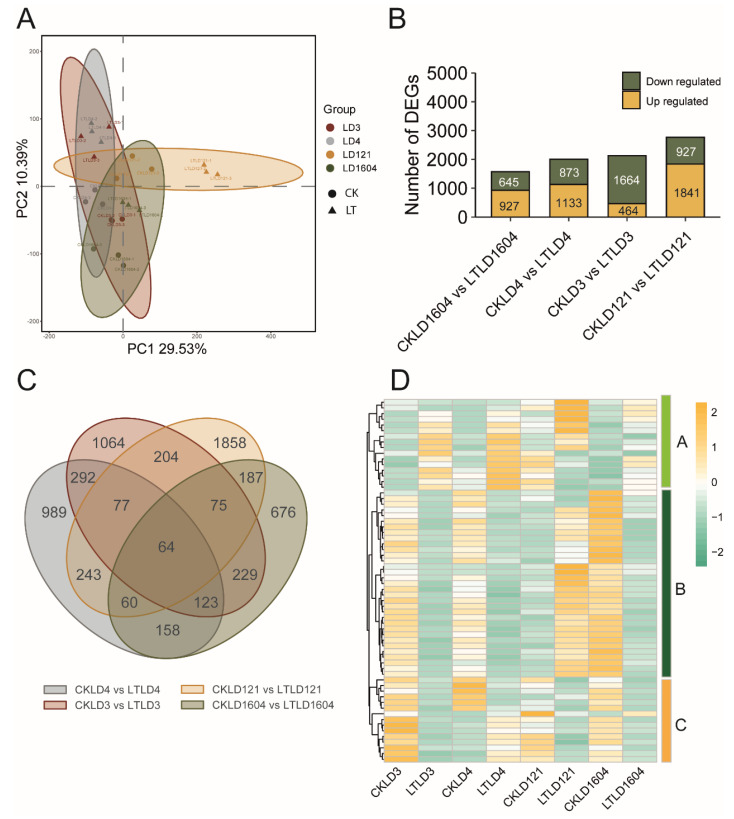
Global transcriptomic profiling of cold-sensitive varieties in response to low-temperature treatment. (**A**) PCA of transcriptome data for CK and LT treatments. (**B**) Bar plot showing the number of DEGs identified in pairwise comparison between CK and LT treatments of the four cold-sensitive varieties. (**C**) Venn diagram illustrating the overlap of DEGs among the four comparison groups. (**D**) Heatmap showing the expression patterns of the 64 DEGs co-expressed across all four cold-sensitive varieties.

PCA was also used to analyze the transcriptomic variation among data of cold-sensitive samples subjected to different treatments. In the PCA plot, the LD3, LD4, and LD1604 samples clustered closely, indicating consistent transcriptomic profiles. Notably, LTLD121 was clearly separated from the other three varieties (Figure 4A), suggesting a distinct transcriptional response to low-temperature stress. A total of 6299 DEGs were identified in paired comparisons between CK and LT treatments across the four sensitive varieties. Specifically, 1572 DEGs were detected between CKLD1604 and LT LD1604 (927 upregulated and 645 downregulated), 2006 between CKLD4 and LT LD4 (1133 upregulated and 873 downregulated), 2128 between CKLD3 and LTLD3 (464 upregulated and 1664 downregulated), and 2768 between CKLD121 and LTLD121 (1841 upregulated and 927 downregulated) (Figure 4B). In addition, Venn diagram analysis of the DEGs showed that 1858, 1,064, 989, and 676 DEGs were uniquely expressed in LD121, LD3, LD4, and LD1604, respectively. Moreover, 64 DEGs were commonly expressed across the four comparison groups. These shared DEGs were classified into three expression modules based on their expression patterns. DEGs in module A were primarily upregulated following LT treatment, particularly in LTLD3, LTLD4, and LTLD121. Module B DEGs were mainly upregulated in CKLD3, CKLD4, and CKLD1604, while a subset showed significant upregulation in LTLD121. In contrast, the genes in Module C were significantly upregulated in CKLD3, CKLD121, and CKLD1604 (Figure 4D). Overall, LD121 displayed a more distinct and specific transcriptomic response to low-temperature treatment compared with the other three cold-sensitive varieties. KEGG enrichment analysis showed that DEGs of the four varieties were co-enriched in several pathways, including metabolic pathways, biosynthesis of secondary metabolites, carbon metabolism, phenylpropanoid biosynthesis, and starch and sucrose metabolism (Figure 3B).

### 3.5. Transcriptomic Regulation of Starch and Sucrose Metabolism Pathways Under Low-Temperature Stress in Cold-Tolerant and Cold-Sensitive Rice Varieties

**Figure 5:**
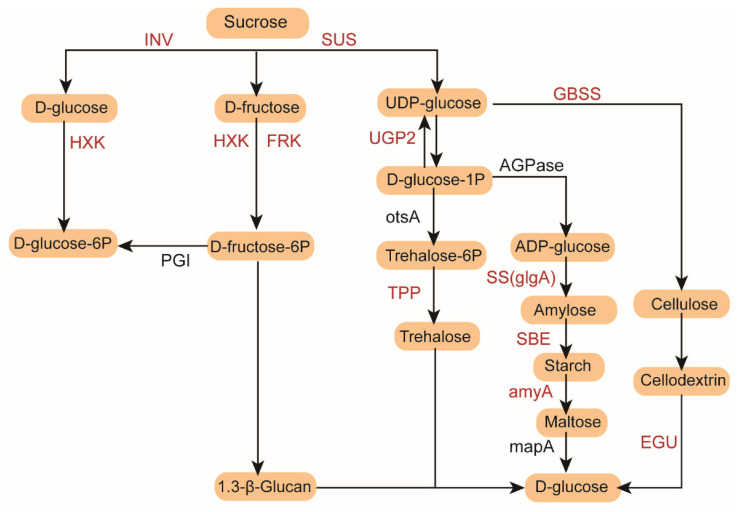

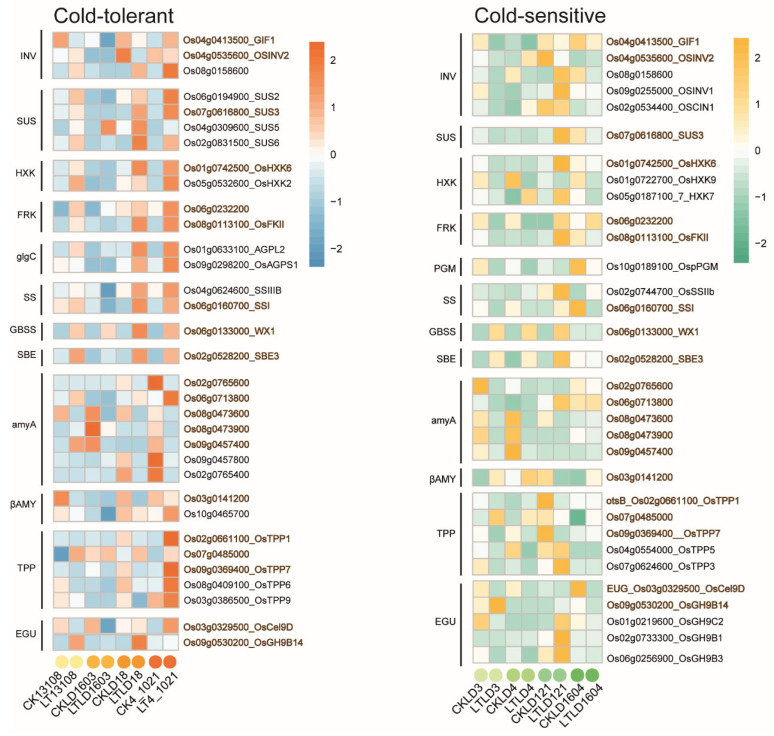
Transcriptomic regulation of starch and sucrose metabolism pathways under low-temperature stress in cold-tolerant and cold-sensitive rice varieties. The schematic diagram depicts key enzymatic steps involved in starch and sucrose metabolism, including pathways for the synthesis and degradation of sugars and polysaccharides such as sucrose, starch, trehalose, and cellulose. Enzymes are labeled in red, and the metabolic intermediates are shown in orange. DEGs corresponding to these enzymes are shown as heatmaps below the pathway for cold-tolerant (**left**) and cold-sensitive (**right**) rice varieties.

Transcriptomic analysis showed that starch and sucrose metabolism was closely related to rice response to low temperature stress. Therefore, we analyzed the expression profile of DEGs related to starch and sucrose metabolism (Figure 5). Invertase (INV), hexokinase (HXK), fructokinase (FRK) and glucose-6-phosphate isomerase (PGI) are key enzymes that catalyze the synthesis of fructose and glucose. In this study, three genes encoding INV, two genes encoding HXK and two genes encoding FRK were identified from the DEGs of cold-tolerant rice varieties. These genes were mainly upregulated in LTLD13108, LTLD18, and LT4-1021 under low-temperature treatment. Additionally, two genes encoding starch synthase (SS), one gene encoding granule-bound starch synthase (GBSS), and 1,4-α-glucan branching enzyme (SBE) were also upregulated in response to low-temperature treatment, particularly in LTLD18 and LT4-1021. Furthermore, seven *amyA* and two *βAMY* genes related to starch degradation were identified. The seven *amyA* genes were significantly upregulated in CK4-1021 but downregulated in all low-temperature-treated samples. Moreover, five genes encoding trehalose-6-phosphate phosphatase (TPP) were highly expressed in LT4-1021. In summary, the DEGs involved in carbohydrate biosynthesis and metabolism were significantly upregulated in LD18 and 4-1021 under LT treatment, indicating that cold-tolerant rice varieties responded to cold stress through starch and sucrose metabolism.

Among the DEGs identified in cold-sensitive rice varieties, five genes encoding INV, three genes encoding HXK, two genes encoding FRK, and one gene encoding SUS were identified. Most of these genes were upregulated in LTLD121. Two genes encoding SS, one gene encoding GBSS, and one gene encoding SBE were also upregulated in LTLD3, LTLD4, and LTLD121 following low-temperature treatment. Five *amyA* genes related to starch degradation were identified, among which Os08g0473600, Os08g0473900, and Os09g0457400 were highly expressed in CKLD3 and CKLD4. In addition, five genes encoding TPP were identified to be highly expressed in CKLD121. In general, DEGs related to starch and sucrose metabolism in cold-sensitive varieties did not show a specific expression pattern that was uniformly upregulated or downregulated in any single variety.

### 3.6. Differential Expression of Transcription Factor (TF) Families in Cold-Tolerant and Cold-Sensitive Rice Under Low-Temperature Stress

**Figure 6:**
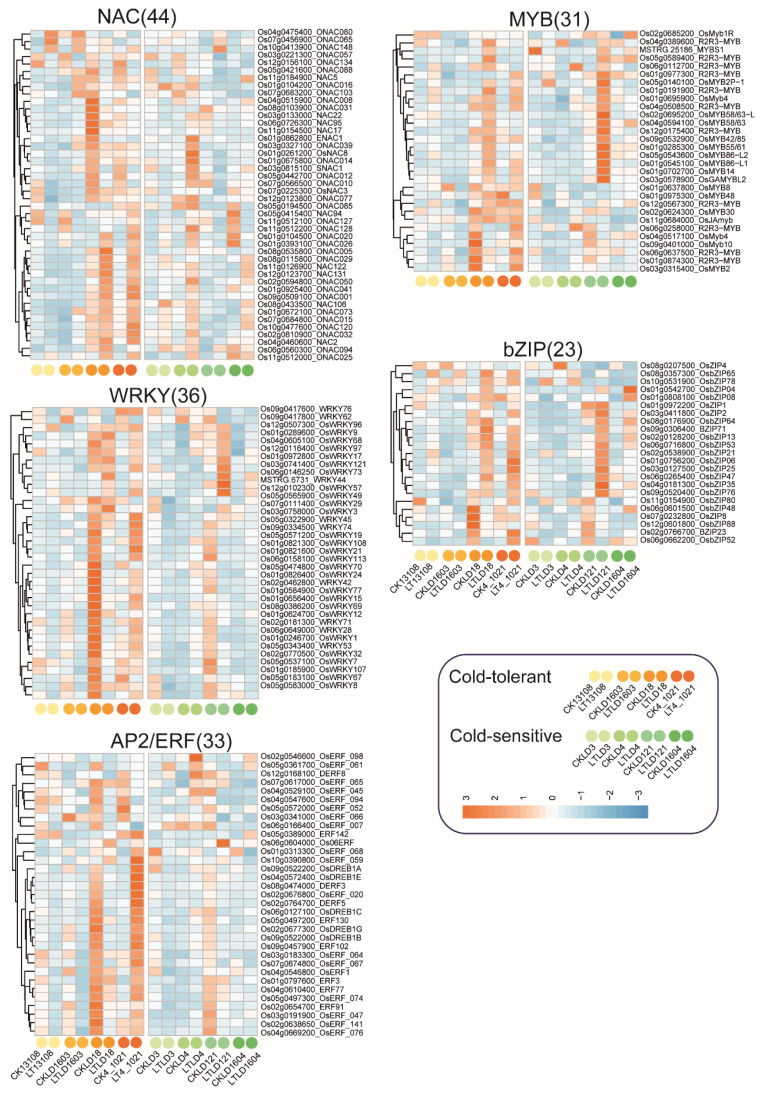
Differential expression of transcription factor (TF) families in cold-tolerant and cold-sensitive rice under low-temperature stress. These TF families include NAC, MYB, WRKY, bZIP, and AP2/ERF.

TFs involved in stress signal transduction and expression regulation play crucial roles in rice under low temperature stress. Among them, members of the NAC, WRKY, AP2/ERF, MYB, and bZIP families are known to participate actively in cold-stress-signaling pathways in rice. In this study, 44 NAC TF genes were identified. In the cold-tolerant variety LD18, gene expression was significantly altered under low-temperature stress, with both upregulation and downregulation observed. These genes also showed increased expression in LT4-1021 and LTLD4 under low-temperature conditions. We also identified 36 WRKY genes and 33 AP2/ERF genes, which were highly expressed in cold-tolerant varieties CKLD18 and LT4-1021. Compared with cold-tolerant varieties, the expression levels of these genes were generally lower in cold-sensitive varieties. Additionally, 31 MYB and 23 bZIP genes were predominantly upregulated in cold-tolerant varieties LTLD18 and LT4-1021, with higher expression levels also detected in the cold-sensitive variety LTLD121 (Figure 6). These findings suggest that rice responds to low-temperature stress by upregulating specific TFs genes. Notably, LD18 and 4-1021 exhibited distinct TF expression patterns before and after low-temperature treatment, reflecting dynamic transcriptional regulation. Among the cold-sensitive varieties, LD121 showed the particularly strong upregulation of various TFs, demonstrating a unique transcriptional response to low-temperature stress.

### 3.7. WGCNA Revealed Key Genes in Cold-Tolerant Rice Under Cold Stress

**Figure 7:**
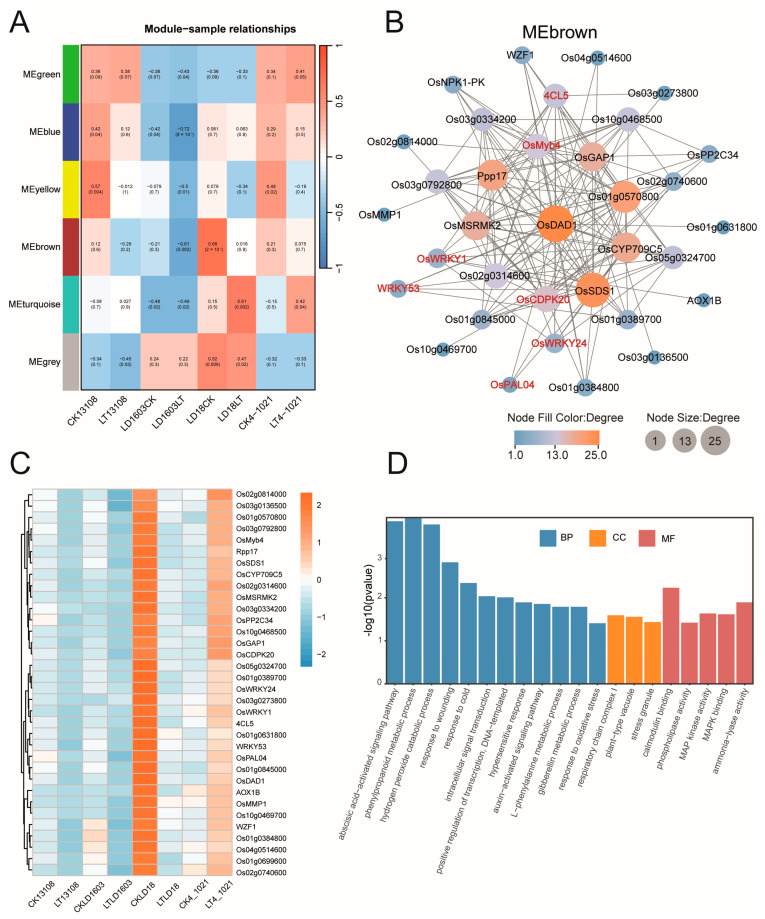
Weighted gene co-expression network analysis (WGCNA) revealed key genes in cold-tolerant rice under cold stress. (**A**) Module–trait correlation heatmap showing the six gene expression modules identified via WGCNA. Each cell shows the Pearson correlation coefficient between gene modules and rice varieties under cold treatment, with red indicating positive correlation and blue indicating negative correlation. The values in parentheses represent the associated *p*-values. (**B**) Co-expression network of hub genes in the MEbrown module. Node size reflects connectivity (degree value). (**C**) Heatmap demonstrating the expression of MEbrown module genes. (**D**) Gene Ontology (GO) enrichment analysis of MEbrown module genes.

To identify key genes involved in the cold response of cold-tolerant rice varieties, we performed WGCNA based on the transcriptome data. The analysis revealed six gene expression modules. Among them, the MEbrown module exhibited the strongest correlation with LD18 (cor = 0.68, *p* = 0.0002) (Figure 7A). Therefore, we selected MEbrown for downstream analysis. Based on network connectivity (weight ≥ 0.15), we visualized the co-expression network and identified core genes within this module. As shown in Figure 7B, several hub genes were located at the center of the MEbrown co-expression network based on degree values, including Os02g0653900 (*OsDAD*), Os01g0864500 (*OsSDS1*), Os01g0570800, and Os01g0841700 (*Ppp17*). In addition, three WRKY genes—Os01g0246700 (*OsWRKY1*), Os05g0343400 (*WRKY53*), and Os01g0826400 (*OsWRKY24*)—and one MYB gene, Os04g0517100 (*OsMyb4*), were also identified in the network. These genes were significantly upregulated in both CKLD18 and LT4-1021 (Figure 7C). Furthermore, genes in the MEbrown module were significantly enriched (*p* < 0.05) in 12 biological process (BP) terms, including the abscisic acid-activated signaling pathway (GO: 0009738), phenylpropanoid metabolic process (GO: 0009698), hydrogen peroxide catabolic process (GO: 1901002), and response to cold (GO: 0009409). This module was also significantly enriched in three cellular components (CCs) and five molecular function (MF) terms (Figure 7D).

## 4. Discussion

Rice endosperm is primarily composed of starch and protein, and its composition is susceptible to abiotic stress [34]. The effects of low temperature on amylose content remain inconsistent across studies. Some studies have reported a decrease in amylose content under low-temperature stress [35,36], while others have observed an increase. Consistent with the findings of Zhang et al. [37] and Shi et al. [38] on *japonica* rice, our study revealed a significant increase in amylose content in both cold-tolerant and cold-sensitive rice varieties after low-temperature treatment. Lu [39] further demonstrated that the effect of low temperature on amylose content varies with rice genotype—decreasing in high-amylose varieties and increasing in low-amylose varieties. In addition, starch content and structure are key determinants of rice texture [40,41]. Low-temperature exposure during the grain filling stage has been shown to negatively affect the taste quality of rice [36,42]. Consistently, our results showed that the taste quality of cold-sensitive varieties declined significantly after low-temperature treatment. Currently, there is no consensus regarding the effect of low temperature on rice protein content: while some studies suggest an increase in protein content upon low-temperature stress during grain filling [37], others report a decrease [42]. In this study, a significant increase in protein content in response to low temperature was observed, which may be attributed to enhanced translocation of soluble proteins from leaves to grains under cold stress. Rice protein primarily consists of albumin, globulin, prolamin, and glutelin, and the proportions of these components can influence both plant response to low temperature and the resulting taste quality of rice. Therefore, future research should focus on quantifying individual protein components in the tested rice varieties to identify which are most responsive to cold stress, and contribute to the observed changes.

In this study, we performed dynamic transcriptome sequencing on rice grains from both cold-tolerant and cold-sensitive rice varieties. A total of 4566 DEGs were identified from the four cold-tolerant varieties. KEGG enrichment analysis showed that these DEGs were mainly concentrated in phenylpropanoid biosynthesis, starch and sucrose metabolism, metabolic pathways, and biosynthesis of secondary metabolites. In cold-sensitive varieties, 6299 DEGs were identified, with enrichment primarily in metabolic pathways, biosynthesis of secondary metabolites, carbon metabolism, phenylpropanoid biosynthesis, and starch and sucrose metabolism. Notably, both cold-tolerant and cold-sensitive varieties were significantly enriched in the starch and sucrose metabolism pathway, suggesting its active role in response to low-temperature stress. Starch and sucrose metabolism provides the essential substrates for grain filling [43]. Studies have shown that low-temperature stress can affect the transcription level of key enzymes in the sucrose metabolism pathway in wheat [44,45], rice [46], and maize [47], significantly altering the accumulation of starch and sugar, and thereby interfering with the grain-filling process [48]. Invertase (INV), hexokinase (HXK), and fructokinase (FRK) are involved in the breakdown of fructose and glucose. In this study, the expression of genes encoding INV, HXK, and FRK was upregulated in cold-tolerant LD18 and 4-1021 under low-temperature stress, which may be attributed to an increase in sucrose concentration and accumulation in the grains. Sucrose synthase (SUS), a key catalytic enzyme for sucrose synthesis and decomposition, also plays an important role in the adaptation of plants to abiotic stresses such as hypoxia and low temperature [44]. We found that *SUS2*, *SUS3*, *SUS5*, and *SUS6* were upregulated under low-temperature treatment in cold-tolerant 13108 and LD18, and in 4-1021, indicating that sucrose synthase played an active role in the regulation of low-temperature stress in rice [49,50]. It is worth noting that the above sucrose-synthesis-related enzyme genes also showed an upward trend in LT-treated LD121, although LD121 was previously identified as a cold-sensitive variety. This observation implies that LD121 may represent a special rice variety, and its potential for cold tolerance warrants further investigation.

TPP is a key enzyme in the trehalose biosynthesis pathway. Under low-temperature (4 °C) stress, the expression of *OsTPP1/2* in rice has been reported to increase by 3–5 times [51]. Our study also found that the *TPP* gene was significantly upregulated in cold-tolerant variety 4-1021 under low-temperature treatment. In addition, studies have shown that overexpression of *OsTPP1* significantly enhances rice tolerance to salt stress under low-temperature stress conditions [51,52,53]. *OsTPP1* promotes the expression of sugar-transport-related genes by inhibiting the accumulation of trehalose hexaphosphate, thereby facilitating sugar accumulation in grains. Sugars can maintain normal pollen development under low-temperature stress, ultimately contributing to improved cold tolerance at the filling stage. The ability of grains to convert sucrose into starch is positively correlated with starch accumulation, as greater conversion efficiency leads to higher starch deposition [54]. In this study, we found that low temperature significantly increased the expression of *SS*, *GBSS*, and *SBE* genes in the grains of cold-tolerant varieties LD18 and 4-1021, indicating that sucrose conversion was less restricted by low-temperature stress in cold-tolerant varieties during grain development. Overall, cold-tolerant rice maintained more stable sucrose input and transformation at low temperature.

Cold stress can rapidly induce the expression of numerous TFs, which, in turn, activate a wide array of downstream cold-responsive genes [55]. Among these, CBF/DREB1 is an AP2-type TF that directly binds to the promoters of downstream cold-responsive genes and regulates their expression, thus mediating plant response to low temperature [56]. In *Arabidopsis thaliana*, the overexpression of OSDREB1A can induce stress-related gene expression and enhance cold stress tolerance [57]. Similarly, in rice, OSDREB1A has been shown to promote extracellular Ca^2+^ influx, increase intracellular Ca^2+^ concentration, and activate low-temperature-stress-related genes, contributing to improved cold tolerance [58]. Consistent with these findings, our study observed the significant upregulation of OsDREB1A expression in the cold-tolerant variety 4-1021 under cold stress.

To elucidate the transcriptional regulatory network and identify key genes involved in cold stress response at the filling stage of cold-tolerant rice varieties, WGCNA was performed on the transcriptome data. The results revealed three WRKY genes in the MEbrown network: OsWRKY1, OsWRKY24, and OsWRKY53. Tang et al. [59] showed that knocking out WRKY53 enhances cold tolerance in multiple rice lines, indicating that OsWRKY53 acts as a negative regulator of cold tolerance at the filling stage. Consistent with this, our study found that OsWRKY53 was highly expressed in the grains of the cold-tolerant variety LD18 under normal temperature conditions (25 °C in the field), but was significantly downregulated after low-temperature treatment, confirming its negative regulatory role. Our results verified the negative regulation of OsWRKY53 on the cold tolerance of rice at the filling stage. In addition, OsMYB4 was identified in the MEbrown module. Previous studies have shown that the overexpression of OsMYB4 in transgenic *Arabidopsis* enhances cold tolerance and affects the expression of various genes in the cold stress response pathway of transgenic plants, suggesting that Osmyb4 is a general switch for cold tolerance [60]. Plants can also respond to cold stress by regulating specific metabolites. Phenylalanine ammonia-lyase genes (PALs) are critical for phenylalanine metabolism and flavonoid biosynthesis. In the MEbrown module, *OsPAL04* was identified as a hub gene. Studies have shown that increased expression of *OsPAL04* enhances plant cold tolerance [61]. In addition, calcium-dependent protein kinase (CDPK/CPK) is a key regulator that transmits calcium (Ca^2+^) signals to cellular stress responses. Studies have shown that CPKs can enhance cold tolerance in plants [62,63]. In rice, *OsCDPK7* [64] and *OsCDPK13* [65] are known to positively regulate both cold and salt tolerance. However, the function of *OsCDPK20*, another CDPK-encoding gene identified in the MEbrown module, has not been characterized. In summary, multiple hub genes identified in cold-tolerant rice varieties under low-temperature stress may act as either positive or negative regulators of cold stress tolerance. These genes serve as promising candidates for future functional studies through gene knockout or overexpression to validate their roles in the cold stress response.

## Data Availability

The sequencing data generated in this study have been deposited in the NCBI Sequence Read Archive (SRA) under the BioProject accession number PRJNA1284649.

## Supplementary Materials

The following supporting information can be downloaded at: https://www.mdpi.com/article/10.3390/genes16080950/s1, Figure S1: Representative images of the eight rice varieties used in this study; Table S1: Taste quality evaluation of 51 rice accessions (2018–2020); Table S2: Quality control and mapping statistics of transcriptome sequencing data across all samples, including raw reads, clean reads, base quality scores, GC content, and mapping rates.
