# Supplementary figures and images for: Transcriptomic Dynamics of Rice Varieties with Differential Cold Tolerance Under Low-Temperature Stress During Grain-Filling Stage
Source: Genes (Basel). 2025 Aug 11;16(8):950. doi: 10.3390/genes16080950 (PMC12385969; doi:10.3390/genes16080950)

**Cold-tolerant**

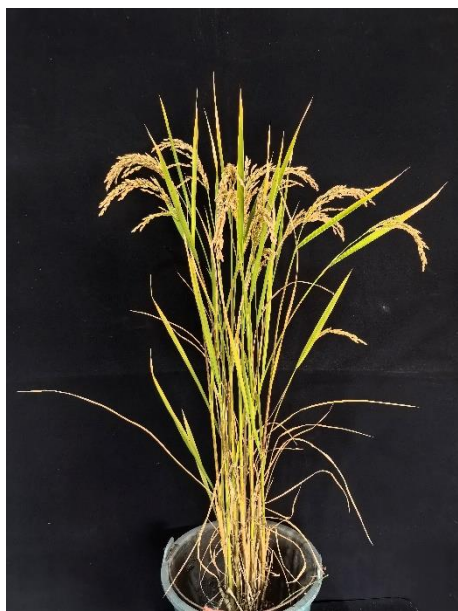

LD1603

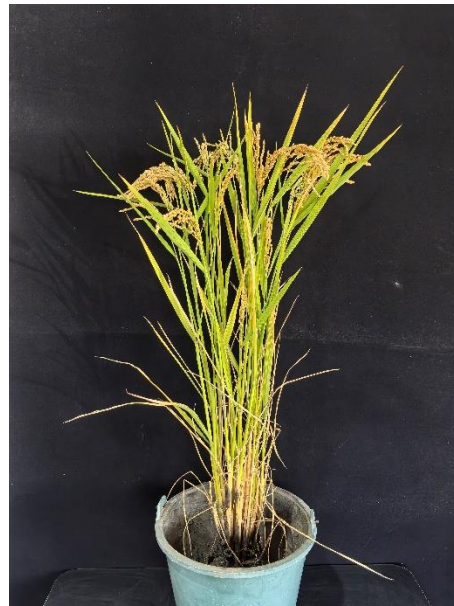

13108

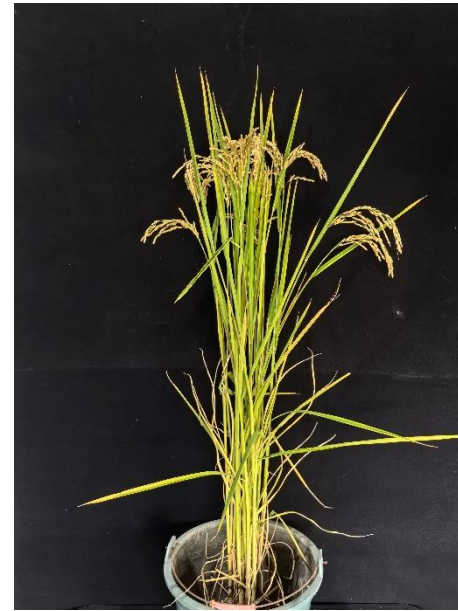

LD18

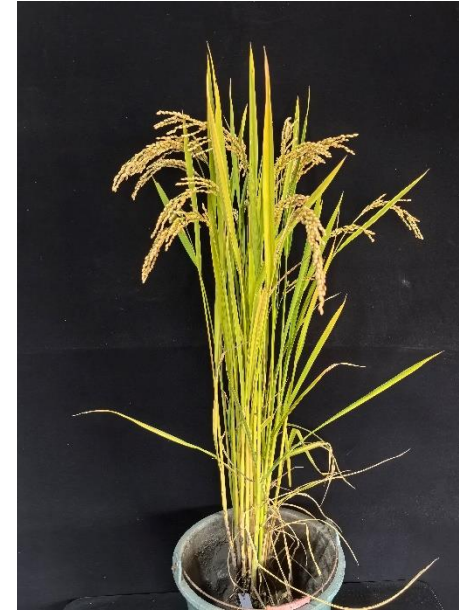

4-1021

**Cold-sensitive**

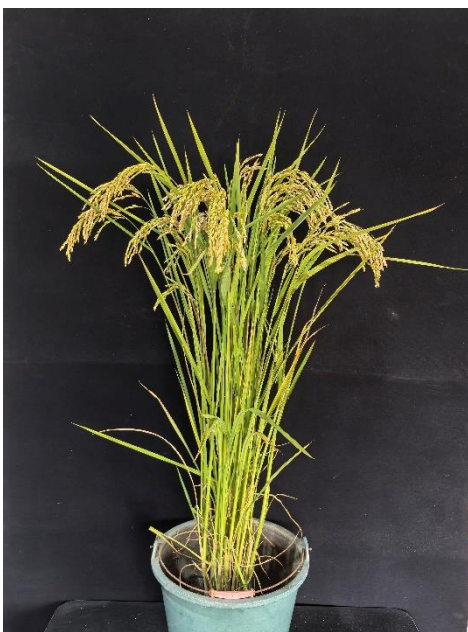

LD3

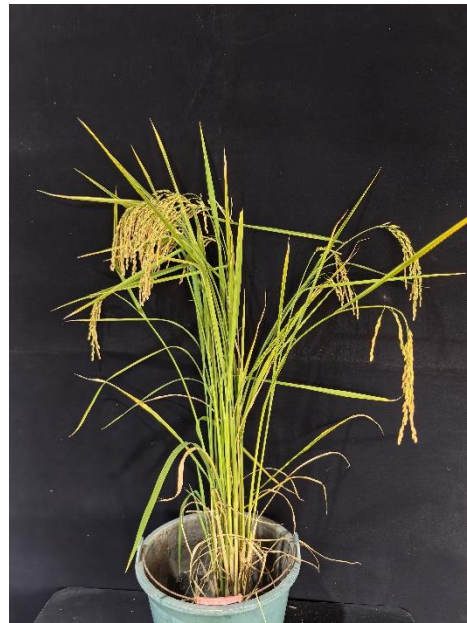

LD4

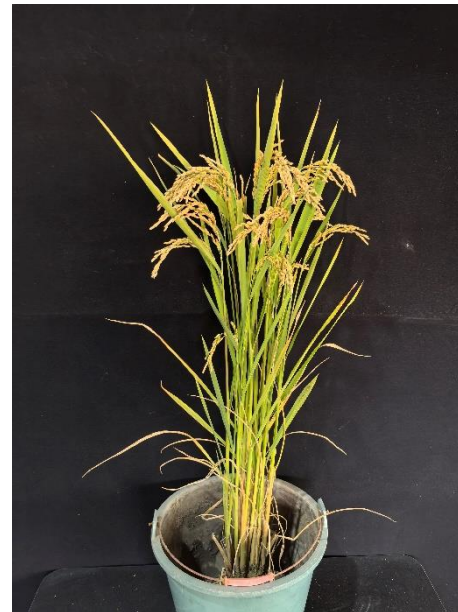

LD121

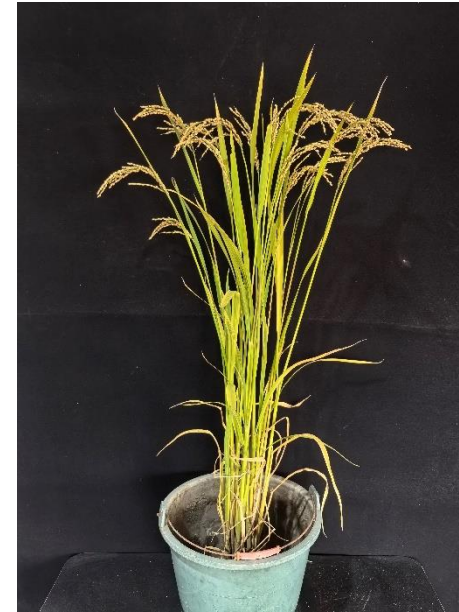

LD1604

Supplement: Supplementary file 1 [file genes-16-00950-s001.zip › Figure S1.pdf]
